# Characterization of the Complete Mitochondrial Genome of *Fischoederius elongatus* Derived from Cows in Shanghai, China

**DOI:** 10.1155/2020/7975948

**Published:** 2020-01-11

**Authors:** Zhaoqing Han, Kun Li, Houqiang Luo, Muhammad Shahzad, Khalid Mehmood

**Affiliations:** ^1^College of Agriculture and Forestry Science, Linyi University, Linyi 276005, China; ^2^College of Veterinary Medicine, Huazhong Agricultural University, Wuhan, China; ^3^College of Animal Science, Wenzhou Vocational College of Science and Technology, Wenzhou 325006, China; ^4^University College of Veterinary & Animal Sciences, The Islamia University of Bahawalpur, Bahawalpur, Pakistan

## Abstract

A study was conducted to reveal the characterization of the complete mitochondrial genome of *Fischoederius elongatus* derived from cows in Shanghai, China. Results indicated that the complete *mt* genome of *F. elongatus* was 14,288 bp and contained 12 protein-coding genes (*cox1-3*, *nad1-6*, *nad4L*, *atp6*, and *cytb*), 22 transfer RNA genes, and two ribosomal RNA genes (l-rRNA and s-rRNA). The overall A + T content of the *mt* genome was 63.83%, and the nucleotide composition was A (19.83%), C (9.75%), G (26.43%), and T (44.00%). A total of 3284 amino acids were encoded by current *F. elongatus* isolate *mt* genome, TTT (Phe) (9.84%) and TTG (Leu) (7.73%) codon were the most frequent amino acids, whereas the ACC (Thr) (0.06%), GCC (Ala) (0.09%), CTC (Leu) (0.09%), and AAC (Asn) (0.09%) codon were the least frequent ones. At the third codon position of *F. elongatus mt* protein genes, T (50.82%) was observed most frequently and C (5.85%) was the least one. The current results can contribute to epidemiology diagnosis, molecular identification, taxonomy, genetic, and drug development researches about this parasite species in cattle.

## 1. Introduction

*Fischoederius elongatus* is a representative of *Paragonimus* genus, which was frequently discovered in ruminants in tropical and subtropical regions [1]. Weight loss and decease of milk production were the common effects of infections caused by *F. elongatus* [2]. Due to the serious economic losses caused by this trematode, a growing trend of attentions was paid towards the *F. elongatus* [3]. According to National Bureau of Statistics of China, population of 89.15 and 297.13 million heads of cattle and sheep, respectively, was estimated in 2018 (http://data.stats.gov.cn/easyquery.htm?cn=C01). In a study, the prevalence of *F. elongatus* infection in cattle and sheep was found to be 50% and 10%, respectively, in Jiangjin, China [4]. However, little knowledge is known about the genetic information of *F. elongatus* in cows in China.

As a maternal inheritance circular genome, mitochondrial genome is popularly utilized in studies for taxonomy and phylogenetic analysis [5, 6]. A higher mutational rate has been observed in *mt* DNA than that of nuclear DNA [7], and due to the alterations in contents of *mt* DNA, it has been reported to be highly related to various diseases [8]. Hundreds of parasitic *mt* genomes are available at NCBI (https://www.ncbi.nlm.nih.gov/nucleotide/); however, there is relatively a lack of data about the *mt* genomes of trematodes. Until now, to the best of our knowledge, only one report is available about the *mt* genome of *F. elongatus* isolated from cattle [3]. The present research herein was aimed to reveal the characterization of the complete *mt* genome of *F. elongatus* derived from cows in Shanghai, China.

## 2. Materials and Methods

### 2.1. Ethical Statement

All procedures adopted in the current research were followed according to the laws, regulations, and guidelines of the Laboratory Animals Research Centre of Hubei province, P. R. China, and the Ethics Committee of Huazhong Agricultural University.

### 2.2. Parasite Collection

Adult trematodes were collected from the cows in Shanghai in 2019. Morphological examination was conducted after extensive washing in 0.9% sodium chloride solution [9]. All the samples were fixed in 75% alcohol (V/V) and kept at −2°C for further utilization as narrated in a previous research [5].

### 2.3. Mitochondrial DNA Sequencing

The extraction of *mt* DNA of *F. elongatus* was performed by employing a commercial Mitochondria Isolation Kit (Sigma-Aldrich, China). The agarose gel electrophoresis method and nanodrop detection were used for the integrity and purity of DNA. All the DNA samples were quantified via a QubitFluorometer (3.0). DNA samples were disrupted into fragments randomly via the ultrasonic method. End repair, A-tailing, index adapter adding, amplification, and purification were performed for library constriction according to the manufacturer's instructions (Illumina). These libraries were sent to commercial sequencing via an Illumina HiSeq X sequencing system at Personalbio in Shanghai, China.

### 2.4. Sequencing Analysis and Genome Annotation

To obtain high accurate sequencing clean data, all the obtained raw reads were filtered with quality score (*Q* < 10) (90%), uncalled bases (“*N*” characters) (>10%), and duplicated sequences. The *mt* genome of *F. elongatus* was assembled via SPAdes v3.11.1 (http://cab.spbu.ru/software/spades/). The *mt* genome assembling and annotation of *F. elongatus* were performed online using the DOGMA tool and MITOS [10, 11]. The circular *mt* genome of the *F. elongatus* genomic map was drawn via OGDraw v1.2 [12].

### 2.5. Nucleotide Variation Analysis

The nucleotide variation of *F. elongatus* between the Tianmen isolate (KM397348.1) and the current isolate was analyzed by employing DnaSp 5.0.

### 2.6. Phylogenetic Analysis

The phylogenetic relationships of *F. elongatus* and other available trematodes were based on *mt* genome using the neighbor-joining method with Kimura two-parameter analysis and bootstrap analysis of 1000 replicates (MEGA 6.0). The available trematodes were *F. elongatus* (KM397348.1), *F. cobboldi* (KX169164.1), *Gastrothylax crumenifer* (KM400624.1), *Paramphistomum cervi* (KT198987.1, KF475773.1), *Calicophoron microbothrioides* (KR337555.1), *Orthocoelium streptocoelium* (KM659177.1), *Explanatum explanatum* (KT198989.1), *Homalogaster paloniae* (KT266674.1, KX169165.1), and *Ogmocotyle* sp. (KR006935.1). The numbers on the branches indicate the percentage of replicates that reproduced the topology for each clad.

## 3. Results and Discussion

**Figure 1:**
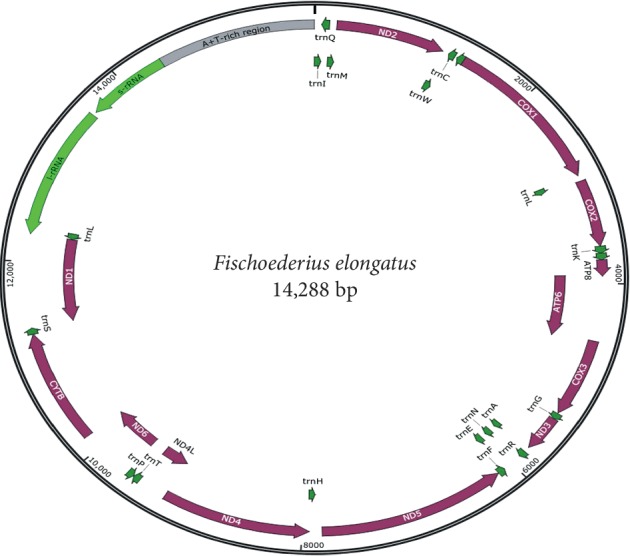
Arrangement of the *mt* genome of *F. elongatus*.

In our study, the complete *mt* genome of the *F. elongatus* isolate was 14,288 bp long (Figure 1), which is longer (by 168 bp) than that of the *F. elongatus* isolated from Tianmen, China (14,120 bp) [3]. The difference may be because of employing different techniques and possibly the genetic prediction error; however, the gene and length are in line with each other. The present sequence of the *mt* genome has been submitted to the GenBank with the Accession number: MN537973. The circular *mt* genome of *F. elongatus* contains 12 protein-coding genes (*cox1-3*, *nad1-6*, *nad4L*, *atp6*, and *cytb*), 22 transfer RNA genes, and two ribosomal RNA genes (*l-rRNA* and *s-rRNA*) (Figure 1, Table 1); however, it lacks *atp8*, which is in line with *F. elongatus* of the Tianmen isolate and *mt* genomes of other trematodes, such as *Gastrothylax crumenifer* and *Paramphistomum cervi* [3, 13, 14]. The protein-coding genes of current *F. elongatus* isolates were transcribed in the same direction, and those genes were assembled in line of *cox3*, *cytb*, *nd4L*, *nd4*, *atp6*, *nd2*, *nd1*, *nd3*, *cox1*, *l-rRNA*, *s-rRNA*, *cox2*, *nad6,* and *nad5* which was in accordance with previously reported results [3, 13, 14] (Figure 1; Table 1).

The overall A + T content of the *mt* genome of the current *F. elongatus* isolate was found to be 63.83%, and the nucleotide composition was A (19.83%), C (9.75%), G (26.43%), and T (44.00%). Moreover, T was the most favored nucleotide, while C was the least common one. These findings are also in accordance with the isolate results of Tianmen [3].

Among the 12 protein genes of the present *F. elongatus* isolate, ATG (5/12) and GTG (5/12) were the most common start codons and TAA (9/12) was the predominant stop codon (Table 1). In current results herein, the 3′-end of genes of *nd1*, *nd3*, *nd4*, *nd5*, *dn6*, *atp6*, *cox3,* and *cox1* was found immediately adjacent to a downstream tRNA gene (Table 1), which was in parallel arrangement with *F. elongatus* Tianmen isolates of *Gastrothylax crumenifer* and *Paramphistomum cervi* [3, 13, 14].

In our study, a total of 3284 amino acids were encoded from the *F. elongatus* isolate *mt* genome excluding the termination codons. All of the 63 possible codons except CGC were found in the *F. elongatus* isolate. TTT (Phe) (9.84%) and TTG (Leu) (7.73%) codon were the most frequent amino acids found, whereas the ACC (Thr) (0.06%), GCC (Ala) (0.09%), CTC (Leu) (0.09%), and AAC (Asn) (0.09%) codon were the least frequent ones uncovered in *F. elongatus* (Table 2). Preferable codons were commonly uncovered with important functional gene regions, as those bias codons with silent sites were found to be related to maximize the translation efficiency [15, 16]. At the third codon position of the current *F. elongatus mt* protein genes, T (50.82%) was the most frequently observed and C (5.85%) was used least frequently ([Supplementary-material supplementary-material-1]). In codons with ≥2 unique bias, the TT codons were noticed at the highest point while CC ones were least found ([Supplementary-material supplementary-material-1]). These interesting results may reveal that *F. elongatus mt* is biased toward utilizing T-rich amino acid codons which are suggestive of the nucleotide bias [17]. However, until now, it is still unclear whether this bias of codon usage contributes to parasite *mt* systems or not [18].

**Figure 2:**
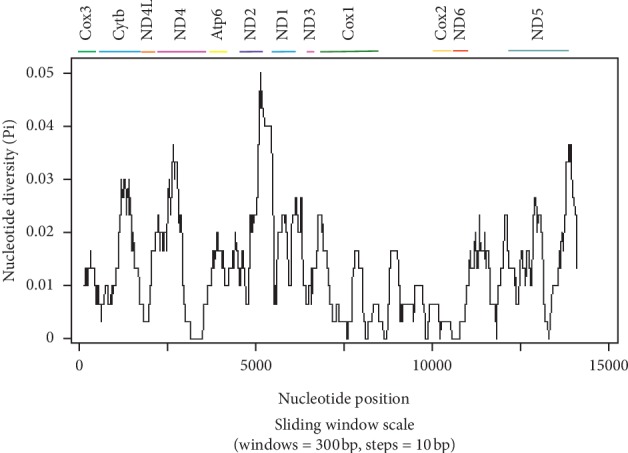
A sliding window analysis of the complete *mt* genome sequences of *F. elongatus* of the current isolate and Tianmen isolate (KM397348.1).

A total of 21 tRNA gene sequences (61–71 bp) and 2 noncoding regions (NCR) (*l-RNA* and *s-RNA*) were found in the *F. elongatus mt* genome (Figure 1, Table 1) depicting one undetected tRNA gene in the current isolate, as other trematodes contain 22 tRNA gene sequences [3, 13, 14]. The L-RNA and s-RNA were found to be located between *cox1* and *cox2* and separated by tRNA-Thr. Though NCR have been commonly reported in trematodes, however, still scarce information were available regarding the function of these special sequences [19]. Two AT-loops (63 and 473 bp) were found in the *F. elongatus mt* genome located between *Cytb* and *Nd4L* and after tRNA-Glu, respectively. By comparing *mt* genome of *F. elongatus* isolated from the cows of Shanghai with 11 available trematodes and employing phylogenetic analysis; the current *F. elongatus* isolates was found highly homologous with the Tianmen isolate (KM397348). The identity between the present isolate and Tianmen isolate has been observed as 98.73% via Nucleotide Blast (https://blast.ncbi.nlm.nih.gov/Blast.cgi?PROGRAM=blastn&PAGE_TYPE=BlastSearch&LINK_LOC=blasthome). Sliding window analysis of *mt* genome of these two *F. elongatus* isolates revealed the nucleotide diversity (Pi) of the 12 protein-coding genes (Figure 2). *Nd2* and *Nd6* were demonstrated to be the highest and at the lowest level of nucleotide variability, respectively (Table 3).

In conclusion, complete *mt* DNA sequences of *F. elongatus* isolated from cows can contribute to the epidemiological diagnosis, molecular identification, taxonomy, genetic and drug development researches about this parasite species [3], and to get benefits for control measures of *Fischoederius* sp. in cattle.

## Figures and Tables

**Table 1:** The *mt* genome of *F. elongatus* isolated from cows using MITOS.

Gene	Position	Length (bp)	Start/stop codon of PCGs	Anticodons
*COX3*	1–645	645	ATG/TAG	
tRNA-His	646–713	68		GTC
*CYTB*	714–1829	1116	ATT/TAA	
AT-loop	1830–1892	63		
*ND4L*	1893–2156	264	ATG/TAG	
*ND4*	2117–3397	1281	GTG/TAA	
tRNA-Gln	3409–3471	63		GTT
tRNA-Phe	3485–3549	65		TTC
tRNA-Met	3549–3612	64		AGA
*ATP6*	3613–4128	516	ATG/TAG	
*ND2*	4133–5008	876	GTG/TAG	
tRNA-Val	5041–5101	61		AAG
tRNA-Ala	5109–5179	71		AAC
tRNA-Asp	5431–5500	70		CAT
*ND1*	5530–6399	870	TTG/TAG	
tRNA-Asn	6419–6484	66		TGG
tRNA-Pro	6489–6552	64		CAG
tRNA-Ile	6554–6616	63		CCG
tRNA-Lys	6623–6687	65		CTG
*ND3*	6701–7048	348	GTG/TAG	
tRNA-Ser	7060–7119	60		GAG
tRNA-Trp	7132–7196	65		AGT
*COX1*	7200–8741	1542	GTG/TAA	
l-rRNA	8575–9865	1291		
tRNA-Thr	8751–8814	64		GAA
s-rRNA	9815–10,603	789		
*COX2*	10,624–11,205	582	ATG/TAG	
*ND6*	11244–11651	408	ATG/TAG	
tRNA-Tyr	11,673–11,737	65		CAG
tRNA-Leu	11,757–11,820	64		TAA
tRNA-Ser	11,822–11,890	69		GTG
tRNA-Leu	11,897–11,961	65		ATA
tRNA-Arg	11,965–12,030	66		TGA
*ND5*	12,031–13,611	1581	GTG/TAG	
tRNA-Gly	13,615–13,679	65		ACG
tRNA-Glu	13,692–13,755	64		TTG
AT-loop	13,756–14,228	473		

**Table 2:** Codons usage of *F. elongatus mt* DNA-encoded proteins.

Amino acid	Codon	Number	Frequency (%)	Amino acid	Codon	Number	Frequency (%)
Phe	TTT	323	9.84	Pro	CCT	34	0.80
Phe	TTC	27	0.82	Pro	CCC	4	0.12
Leu	TTA	163	4.96	Pro	CCA	11	0.26
Leu	TTG	254	7.73	Pro	CCG	8	0.19
Leu	CTT	41	1.25	Thr	ACT	52	1.58
Leu	CTC	3	0.09	Thr	ACC	2	0.06
Leu	CTA	17	0.52	Thr	ACA	18	0.55
Leu	CTG	25	0.76	Thr	ACG	16	0.49
IIe	ATT	128	3.90	Ala	GCT	98	2.98
IIe	ATC	5	0.15	Ala	GCC	3	0.09
IIe	ATA	73	2.22	Ala	GCA	14	0.43
Val	GTT	176	5.36	Ala	GCG	31	5.18
Val	GTC	13	0.40	Tyr	TAT	170	5.18
Val	GTA	56	1.71	Tyr	TAC	8	0.24
Val	GTG	167	5.09	His	CAT	42	1.28
Ser	TCT	116	3.53	His	CAC	6	0.18
Ser	TCC	8	0.24	Gln	CAA	13	0.40
Ser	TCA	23	0.70	Gln	CAG	25	0.76
Ser	TCG	32	0.97	Asn	AAT	55	1.67
Ser	AGT	80	2.44	Asn	AAC	3	0.09
Ser	AGC	13	0.40	Arg	CGT	45	1.37
Trp	TGG	74	2.25	Arg	CGC	0	0
Lys	AAA	20	0.61	Arg	CGA	6	0.18
Lys	AAG	52	1.58	Arg	CGG	11	0.33
Asp	GAT	61	1.86	Arg	AGA	30	0.91
Asp	GAC	4	0.12	Arg	AGG	36	1.10
Glu	GAA	20	0.61	Gly	GGT	160	4.87
Glu	GAG	64	1.85	Gly	GGC	20	0.61
Cys	TGT	116	3.53	Gly	GGA	24	0.73
Cys	TGC	7	0.21	Gly	GGG	50	1.52
Met	ATG	3.11	2.48				

**Table 3:** Multidomain analysis of the complete *mt* genome sequences of *F. elongatus* of the current isolate and Tianmen isolate (KM397348.1).

Region	*n*	Sites	Net sites	*S*	Eta	Hap	Hd	VarHd	Pi
1–14,228	2	14,228	14,120	181	181	2	1.000	0.25000	0.01282

## Data Availability

The data used to support the findings of this study are available from the corresponding author upon request.

## Supplementary Materials

Supplementary Materials Figure S1: statistics of the third position of codons bias usage of F. elongatus mt DNA-encoded proteins. Figure S2: codons bias usage of F. elongatus mt DNA-encoded proteins. .
