# Supplementary material for: Characterization of the Complete Mitochondrial Genome of Fischoederius elongatus Derived from Cows in Shanghai, China
Source: Biomed Res Int. 2020 Jan 11;2020:7975948. doi: 10.1155/2020/7975948 (PMC6982361; doi:10.1155/2020/7975948)
Supplement: Supplementary Materials — Figure S1: statistics of the third position of codons bias usage of F. elongatus mt DNA-encoded proteins. Figure S2: codons bias usage of F. elongatus mt DNA-encoded proteins. . [file 7975948.f1.docx]

Figure S1: Statistics the third position of codons bias usage of *F. elongatus* *mt* DNA encoded proteins

Figure S2: Codons bias usage of *F. elongatus* *mt* DNA encoded proteins
